# Following the dynamics of matter with femtosecond precision using the X-ray streaking method

**DOI:** 10.1038/srep07644

**Published:** 2015-01-06

**Authors:** C. David, P. Karvinen, M. Sikorski, S. Song, I. Vartiainen, C. J. Milne, A. Mozzanica, Y. Kayser, A. Diaz, I. Mohacsi, G. A. Carini, S. Herrmann, E. Färm, M. Ritala, D. M. Fritz, A. Robert

**Affiliations:** 1 Paul Scherrer Institut, CH-5232 Villigen, Switzerland; 2 SLAC National Accelerator Laboratory, Menlo Park, CA 94025, USA; 3 Department of Chemistry, University of Helsinki, Helsinki FI-00014, Finland

## Abstract

X-ray Free Electron Lasers (FELs) can produce extremely intense and very short pulses, down to below 10 femtoseconds (fs). Among the key applications are ultrafast time-resolved studies of dynamics of matter by observing responses to fast excitation pulses in a pump-probe manner. Detectors with sufficient time resolution for observing these processes are not available. Therefore, such experiments typically measure a sample's full dynamics by repeating multiple pump-probe cycles at different delay times. This conventional method assumes that the sample returns to an identical or very similar state after each cycle. Here we describe a novel approach that can provide a time trace of responses following a single excitation pulse, jitter-free, with fs timing precision. We demonstrate, in an X-ray diffraction experiment, how it can be applied to the investigation of ultrafast irreversible processes.

The understanding of phenomena in matter often relies on the investigation of its *structure*, meaning its atomic composition, and its *dynamics* describing how the structure undergoes changes. Many fundamental dynamic processes, such as the formation or breaking of chemical bonds, are related to the motion of atoms. The time scales of the vibrational movement of atoms typically lie in the sub-picosecond range. The advent of femtosecond lasers has provided a powerful tool for the study of transient matter. In pump-probe experiments they are used to push a system out of equilibrium using a pulsed excitation (pump) and the subsequent relaxation dynamics are measured by applying a second pulse to determine the status of a particular property of the sample after a given delay (probe). With the advent of suitable sources such as Laser Plasmas^1^,^2^, High-Harmonic Generation^3^, Femto-Slicing^4^, and X-ray Free-Electron Lasers^5^,^6^,^7^, X-ray probe pulses are receiving increasing attention, as X-rays offer a rich variety of resonantly enhanced contrast mechanisms to reveal element-specific and even chemical information. X-ray based spectroscopic methods applied on a femtosecond time scale^8^ can also directly interrogate local changes of electronic states. When using multi-keV or hard X-rays, the structural dynamics of matter can be probed by diffraction experiments^1^,^2^,^9^,^10^.

All these experiments measure one particular delay after the arrival of the pump pulse at a time. Thus, these techniques are essentially limited to the study of reversible processes that reproducibly return to their ground state after each pump-probe cycle, or they require an identical, fresh sample for each shot, with identical orientation in the case of diffraction experiments. This has hindered pump-probe studies of highly excited materials at near-solid densities as they reach local thermal equilibrium: the so-called “warm dense matter” regime^11^. Since these very high-energy density experiments permanently damage the samples with each shot, and constant supply of fresh identical samples is only possible in some experiments, it is most useful to find a way to get complete time traces of the response from a single X-ray pump pulse.

Another important limitation for ultrafast time-resolved experiments at X-ray FEL sources has been the loss in time resolution due to the timing jitter between the pump and probe pulses. Typically, the external pump lasers are synchronized to the X-ray FEL via radiofrequency phase-locking, resulting in short term jitter of more than 100 fs^12^, and long term drifts in the ps range. The development of more advanced techniques, e.g. exploiting the terahertz emission from the FEL undulator^13^ or the signal from electron bunch monitors^14^ for the stabilization of the X-ray and optical pulses, is a topic of intense research. A pragmatic and robust method involves monitoring the relative time delay on a per-shot basis and re-sorting the data accordingly. This approach can reduce timing errors to below 10 fs^15^, but it obviously requires data sets collected using many pump pulses and is therefore of limited use for single-shot experiments. A fundamental way to avoid timing jitter between pulses is to split them from the same parent pulse and to control their temporal separation by a delay line. Several such instruments based on mirrors^16^,^17^ and Bragg crystals^18^,^19^ have been developed for soft and hard X-ray FEL radiation, respectively. However, both approaches only provide one delay time per X-ray pulse.

We pursue a split-and-delay approach based on diffraction gratings as shown in Figure 1. A set of beam splitter gratings S_n_ with different periods *p_n_* diffracts a small fraction of the incoming radiation into a fan of beams. A second set of gratings R_n_ is positioned half way between S_n_ and the sample to recombine the diffracted beams with the direct, undiffracted beam at the sample position. For this purpose, the recombiner periods *q_n_* must be half the period *p_n_* of the corresponding S_n_ grating. The deflection *Δx_n_ = aλ/2p_n_* in the recombiner plane with respect to the undiffracted beam results in a delay *Δt_n_* of 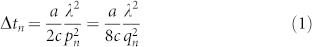
where *a* denotes the distance between S_n_ and the sample, *c* the speed of light, and *λ* the X-ray wavelength. For weak S_n_ gratings - meaning gratings of low diffraction efficiency - most of the intensity remains in the undiffracted beam, which can be used as a pump pulse to excite the sample. This pulse is followed by a series of probe pulses, each a diffracted beam, with delays that are precisely defined by the geometrical parameters and not subject to any pump-probe jitter. The probe beams diverge again downstream of the sample, and a streak of delayed probe pulses can be recorded on a detector array, analogously to commonly used electron streak cameras. No time resolving detector is required.

The positive and negative diffraction orders of each splitter grating S_n_ create a symmetric pair of beams propagating towards the recombiner gratings R_n_. In our set-up depicted in Figure 1, the beams diffracted in the upward direction are deflected by the corresponding R_n_ gratings to probe the pumped region of the sample. However, the beams diffracted downwards are deflected by R_n_ gratings having a slightly larger period, in order to hit the sample at a slightly different position than the pump beam and the probe beams (see inset of Figure 1). The Bragg reflection of these beams can be used to provide the unpumped response for the very same shot as a reference.

In addition to the above-mentioned intrinsic absence of jitter, the availability of several probe beams, and the possibility to record the unpumped response, the experimental setup described here has several particular properties, that make it robust and easy to use at X-ray FEL sources:

- As the R_n_ gratings are placed half-way between the S_n_ gratings and the sample, the set-up is achromatic in the sense that it can accept the full range of photon energies of the self-amplified spontaneous emission (SASE) without losing the intersection point of the pump and probe beams.
- Contrary to set-ups based on reflective optics, the use of transmission gratings makes the set-up insensitive to mechanical drift and vibrations. The deflection angles of the beams are determined by the grating periods, they are not affected by lateral displacements of the gratings, and are very tolerant with respect to changes of the incidence angle onto the gratings.
- Due to the use of two gratings in each delayed beam, the pulse fronts remain parallel to that of the pump beam. This avoids the pulse stretching effect often encountered when using diffractive optics^20^.

We implemented such an experiment at the XCS instrument of the Linac Coherent Light Source (LCLS)^21^, operated at 4.5 keV photon energy and 40 fs pulse length. The essential design parameters are listed in Table 1. The set-up comprises each 15 delayed probe and reference channels with delays spanning over more than a picosecond. As can be seen from eq. 1, the time delays scale linearly with the length of the setup *a*, and inverse to the square of the grating periods. In order to achieve delays of up to 1.277 ps at 4.5 keV photon energy, we chose *a* to be 12.2 m, limited by the dimensions of the experimental hutch. In spite of this large distance, the required periods *q_n_* need to be as small as 17.4 nm, which is close to the fabrication limits of nanolithographic techniques. Moreover, the grating periods need to be exact within extremely narrow tolerances. Furthermore, due to their nanoscale dimensions, the gratings for long delays could only be made with shallow line profiles, making the gratings and thus the corresponding channels very inefficient, see Table 1. More information on the grating fabrication tolerances and a possible route to improve the channel efficiency is given in the methods section. It should be noted at this point that the maximum delay demonstrated in the described experiment does not represent a fundamental limit. As the delay scales proportionally with the sqaure of the X-ray wavelength (see eq. 1), a delay range of more than 11 ps could be covered at 1.5 keV photon energy using the same grating periods *p_n_* and *q_n_* and the same S_n_-to-sample length *a*.

The multiple split-and-delay line was used in the scattering geometry shown in Figure 1 to record the Bragg reflection from a Bismuth <111> crystal. We chose a scattering geometry in the horizontal plane in order to ensure that the delayed beams, which are incident at varying vertical angles, all fulfilled the Bragg condition. Figure 2 displays averaged and single shot data of the Bragg-reflected intensities in the case where the direct pump beam was blocked using the attenuator near the R_n_ plane. Although the signal level drops rapidly with increasing delay, even resulting in several “dead” channels, we can clearly observe the delayed streaks of probe and reference pulses. In particular, it should be noted that the 15^th^ channel can still be disinguished from the detector noise level even for the single shot recordings.

The time delays depend on the grating periods and grating distances, both of which can be determined with a relative accuracy of better than 10^−3^. The dominating uncertainty of the delays is given by the fact that, according to eq. 1, the path length and therefore the delay is wavelength dependent. At LCLS the beam is subject to a 0.5% shot-to-shot wavelength jitter related to fluctuations of the accelerator energy^4^. As the latter is monitored for each shot, its effect on the delay is known. The relative spectral width of the individual pulses is limited by the SASE process to *δλ/λ* ≈ 0.2%^6^, resulting in *δt/Δt* = *2δλ/λ* = 0.4%. The timing uncertainty *δt_n_* is only a few fs even for the longest delays (see Table 1), which is well below the X-ray FEL pulse length itself.

The motivation for using Bi <111> as the sample in these diffraction experiments was to investigate whether phonon oscillations could be observed. Their effect on the Bragg reflectivity of Bismuth has been studied in a number of experiments using infra-red pump lasers for excitation^1^,^8^,^9^. We collected the X-ray streaking signal for several thousand pump events at various pump levels between 1 × 10^9^ W/cm^2^ and just below the damage threshold which was found to be at 2 × 10^12^ W/cm^2^. We tried a variety of experimental settings regarding the pump and probe spot sizes and found no evidence of any oscillatory behaviour in the probe streak. It is unclear whether the contrast of the phonon oscillation signal was too low to be distinguished from shot-to-shot fluctuations of the measured signals or whether no phonon oscillations can be excited with multi-keV X-rays at pump fluences below the damage threshold. The latter explanation is plausible in view of the fact that IR-pumped measurements on phonon oscillations in Bi typically require pump intensities within a factor of two from the stability limit^1^,^9^.

It is expected that the Bragg reflectivity decreases rapidly once the crystal lattice of the sample disorders for pump levels above the damage threshold. We can follow the dynamics of this effect as displayed in Figure 3. In the unpumped case one can observe the same probe and reference streak as in Figure 2, though recorded using a 2-dimensional pixel detector. The three single-shot measurements were performed at a pump fluence far beyond the damage threshold. The probe streak measurement dies out with a decay time of about 50–70 fs, which is somewhat longer than the nominal pump pulse length of 40 fs. The order of the atomic lattice obviously vanishes within a few tens of fs, which is consistent with observations by ultra-fast electron diffraction^22^. The fact that the reference streak signal remains constant proves that the change in Bragg reflectivity is limited to the pumped region. A more precise observation of the lattice dynamics would require a systematic variation of the pump fluence, which was not possible during the available beam time.

These very first X-ray streaking measurements are unique, as they directly show the evolution of the femtosecond response of a sample following a single, destructive pump pulse, free of timing jitter. The presented experimental set-up can be further improved in particular with respect to the channel efficiency and shot-to-shot fluctuations, as described in the methods section.

A variety of novel experiments could be performed by X-ray streaking. One could, for example, use protein crystals as a sample to directly determine the required pulse length in serial nano-crystallography at X-ray FELs^23^. The damage mechanisms and time scales are of high relevance in structural biology and have so far only been investigated by calculations and indirect measurements^24^. Changes in the X-ray absorption on the femtosecond scale could be observed using the multiple split-and-delay line by placing sample and detector in a transmission geometry. Such measurements could reveal the dynamics of processes following multiple core-shell ionization processes or the formation and dissociation of chemical bonds. In this context, the method could also be used with an external pump laser or other excitation mechanisms. This addition would greatly increase the variety of accessible phenomena, and even though it means losing intrinsic timing with respect to the excitation event, the relative timing of the response would still be accurate.

In summary, we have developed a novel technique for X-ray pump-probe measurements that can provide a series of probe pulses for each pump event. The probe beams have different delays with respect of the excitation pulse, which are intrinsically free of timing jitter, and accurate to the femtosecond level. The technique also provides reference pulses with the same delays that provide information of the unpumped sample for normalization purposes. In a demonstrator experiment at 4.5 keV photon energy, we have presented single-shot measurements of the Bragg reflectivity of a Bismuth crystal pumped with a fluence level far beyond the damage threshold. We found that the reflectivity decays within a few tens of femtoseconds, indicating a fast loss of order in the crystal structure. This unique type of ultra-fast measurements opens up a new path towards the investigation of matter subject to extreme excitation levels that cannot easily be investigated by conventional pump-probe techniques requiring many repetitions of pump-probe cycles at different delay values.

## Methods

### Grating fabrication

All gratings were generated using a 100 keV electron-beam writer (Vistec EBPG5000Plus). As the S_n_ gratings were subject to the full LCLS beam, they were made from polished 10 μm thick diamond membranes (Diamond Materials GmbH) to avoid beam damage. Details on the fabrication of diamond diffractive X-ray optics and the radiation hardness in X-ray FEL beams can be found elsewhere^25^. Each diamond grating had an area of 1 mm × 1 mm to accept the full LCLS beam. As the R_n_ gratings only received much lower fluence they could be made on silicon nitride membranes. In analogy to a process for the fabrication of diffractive lenses^26^, we used a line doubling procedure based on the coating with Iridium using an Atomic Layer Deposition (ALD) process^27^. The R_n_ gratings were 500 μm × 500 μm in size.

The diffraction efficiency of all gratings was measured with synchrotron radiation at the cSAXS station of the Swiss Light Source. The channel efficiencies listed in Table 1 are the efficiency products of both gratings in each channel.

### Experimental setup

The experiments were performed at the XCS instrument of LCLS. To reduce absorption losses all components except for the sample and the detectors were placed inside helium-filled enclosures, separated from the beam line vacuum by a 100 μm thick diamond window. The attenuation of the setup was 88% up to the sample and another 40% between the sample and the detectors placed 1.6 m further downstream.

**Figure 1:**
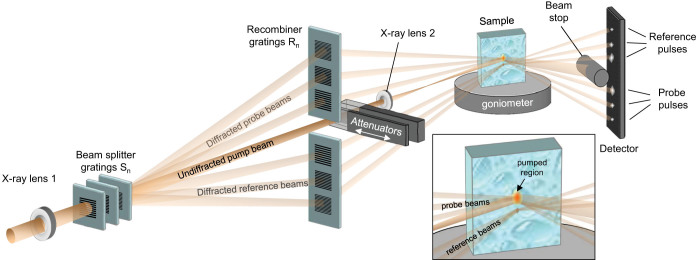
X-ray streaking principle. Diffraction gratings are used to create a multiple split-and-delay line. The upstream X-ray lens 1 focuses the XFEL beam onto the sample. The undiffracted (direct) beam serves as a pump, and can be attenuated and focused independently by X-ray lens 2. The beams diffracted upwards by the splitter gratings S_n_ are redirected towards the sample by the recombiner gratings R_n_, and probe the pumped sample region with defined delays. The beams diffracted downwards by S_n_ are steered to a region of the sample that is 100 μm below the pump beam (see inset) to provide reference signals of the unpumped response on the very same shot. All beams are recorded separately on a detector array. Only three delayed beam pairs are shown for simplicity. The sample's scattering plane is chosen perpendicular to that of the gratings, in order to minimize coupling of the scattering angles.

We used the full SASE emission from LCLS at 4.5 keV photon energy, 2 mJ pulse energy and 40 fs pulse length throughout the experiments. The delayed beams were focused onto the sample by a Beryllium refractive lens (see Fig. 1, X-ray lens 1) giving a spot size close to the diffraction limit of ≈ 1 μm. The size of the direct beam was increased to ≈30 μm by an additional refractive lens (see Fig. 1, X-ray lens 2) to facilitate overlap of the pump and the probe beams. In order to achieve a positioning accuracy of the probe beams with respect to the pump beam of 10 μm or better, the grating diffraction angles need to be precise to better than ≈1 μrad, requiring the grating periods to have a relative accuracy of better than ≈10^−4^. For the finest R_n_ grating this means that its pitch of *q_n_* = 17.391 nm needs to be accurate within ≈0.002 nm.

The alignment of the probe beams and the reference beams with respect to the pump beam was performed by placing a high resolution (~2 μm) X-ray camera in the sample position, and tuning the rotation angles of the S_n_ gratings around the optical axis as well as the distances between the gratings and the sample. A spatial overlap of clearly better than 10 μm could be obtained.

The Bismuth <111> crystal consisted of a cleaved bulk sample (by Mateck GmbH) providing single crystal domains of several mm in size. The Bragg angle was 20.15°, the mosaic spread of the reflections was typically 0.1 mrad, resulting in an accepted bandwidth of about 10 eV, which matches the bandwidth of a single SASE pulse (*δE/E* ≈ 0.2%, *δE* ≈ 9 eV). The damage threshold of Bi was found to be at ≈2 × 10^12^ W/cm^2^.

### Data acquisition and data treatment

**Figure 2:**
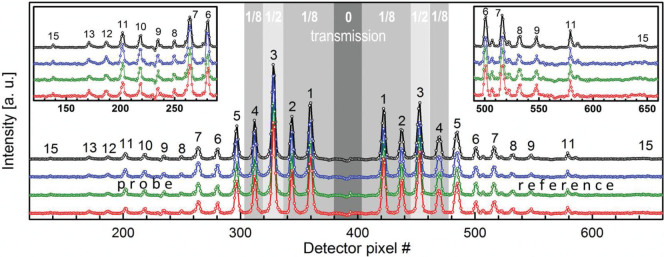
X-ray diffraction data of 15 pairs of delayed probe and reference pulses. The data were taken with the set-up sketched in Fig. 1 using a Bi <111> crystal as sample. The black curve shows the average over twelve XFEL shots, while the coloured curves are single shot data. The curves are shown on a linear scale and are offset in the vertical direction for clarity. The grey regions mark the channels that were reduced in transmission by additional attenuators (not shown in Fig. 1) on the R_n_ gratings in order to make better use of the dynamic range of the strip detector. The direct pump beam was completely blocked. The insets show probe and reference signals for long delays (n ≥ 6, Δt ≥ 243 fs).

For the data shown in Fig. 2, we used a Gotthard strip detector^28^ with 50 μm pitch. A series of 10.000 shots was acquired at 120 Hz repetition rate, while monitoring the accelerator energy and the photon pulse intensity for each shot. We found that the relative peak heights varied substantially from shot to shot, and that this variation is clearly correlated with the accelerator energy and pulse intensity. The data displayed in Fig. 2 were selected to have similar accelerator energies and pulse energies close to the mean values of the series, and therefore exhibit very similar peak heights.

**Figure 3:**
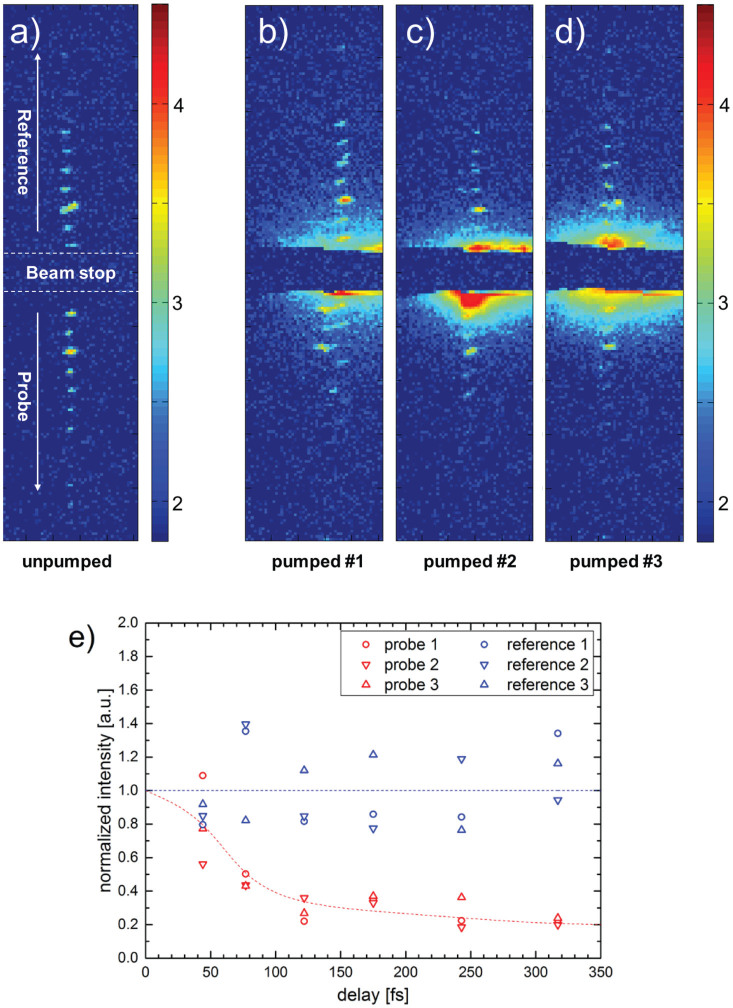
Single-shot time resolved X-ray diffraction measurements. The experimental geometry is the one depicted in Fig. 1, however using a 2-dimensional pixel detector. The centre of the detector is protected by a beam stop. Panels (a)–(d) show the signal of the probe and reference beams on a logarithmic scale (log10). Panel (a) was recorded with the pump beam blocked upstream of the sample, panels (b)–(d) display three single shot measurements at the full pump power density of ≈3 × 10^14^ W/cm^2^, meaning that no attenuators were used in the pump beam. Panel (e) shows the intensities integrated over the spots of (b)–(d), normalized with the corresponding intensities of (a), versus delay time. The probe pulses decay due to the destruction of the Bi crystal lattice, whereas the reference signals remain constant. The dashed lines merely serve to guide the eye and do not represent experimental data.

The data shown in Fig. 3 were collected with a CS-PAD 140 k detector module^29^ with 110 μm × 110 μm pixel size. The data were taken in single shot mode in order to choose a fresh sample region after every shot. The background caused by the flare of the pump beam was subtracted from the delayed probe and reference peaks. The remaining intensity was integrated over the delayed spots and normalized with the integral over the corresponding spots of the unpumped shot. No data binning according to accelerator energy and pulse intensity was applied due to the small number of single-shot experiments. This is the main cause for the fairly large scatter of the data points.

### A route for improvement

Two main limitations of the described set-up are due to the grating performance: (i) The low efficiencies at high channel numbers needs to be enhanced by improving the grating efficiencies. For such weak gratings the efficiency scales with the square of the height of the grating lines. As the channel efficiency is determined by the efficiency product of both corresponding gratings, already a doubling of the structure height in each grating would lead to a channel efficiency increase by a factor of 16. Such an increase in structure height could for example be achieved by using the gratings in a tilted geometry^30^. (ii) The strong shot-to-shot fluctuation of the relative peak heights can be explained by a fluctuating illumination of the gratings. As these devices have inhomogeneous efficiency distributions across their area, this will translate into fluctuations of the channel efficiencies. The correlation with the accelerator energy and thus with the photon energy is due to the corresponding changes in S_n_ diffraction angle, causing the beam to illuminate a different part of - or even partly miss - the R_n_ gratings. This problem must be addressed by fabricating larger and more homogeneous gratings.

## Author Contributions

The experiment was conceived by C.D. and P.K. The experimental hardware, including the diffraction gratings, was developed, fabricated, and characterised by C.D., P.K., M.S., I.M., G.C., S.H., A.M., A.D., E.F. and M.R. The measurements at LCLS were carried out by C.D., P.K., M.S., S.S., I.V., C.J.M., A.M., Y.K., G.C., S.H., D.M.F. and A.R. The LCLS data were analysed by P.K. with contributions from A.R. The manuscript was written by C.D. with extensive contributions from P.K. and suggestions from all other authors.

## Figures and Tables

**Table 1:** Parameters and properties of the multiple split-and-delay line. 15 probe and reference channel pairs were realized in the geometry shown in Figure 1 for 4.5 keV photon energy and a S_n_-to-sample distance of *a* = 12.2 m. The recombiner grating pitches *q_n_* and *q'_n_* refer to the probe and reference channels, respectively. The channel efficiencies *η_n_* and *η'_n_* of the probe and reference channels are the products of the diffraction efficiencies of the two gratings in each channel. They represent the relative channel intensities compared to the direct pump beam intensity when no attenuators are used. *Δt_n_* denotes the resulting delay with respect to the direct pump beam, *δt_n_* is the chromatic delay uncertainty calculated for a 0.2% relative energy bandwidth.

Channel number *n*	1	2	3	4	5	6	7	8	9	10	11	12	13	14	15
Grating period *q_n_ [nm]*	141.364	93.869	70.682	56.196	46.942	39.843	34.908	31.295	28.103	25.467	23.250	21.528	19.952	18.573	17.391
Grating period *q'_n_ [nm]*	142.564	94.396	70.981	56.384	47.074	39.938	34.981	31.353	28.150	25.505	23.282	21.556	19.976	18.593	17.409
Deflection *Δx_n_ [mm]*	6	9	12	15	18	21	24	27	30	33	36	39	42	45	48
Channel efficiency *η_n_*	4.4E-4	2.0E-4	3.0E-5	2.4E-4	1.4E-5	9.8E-6	1.4E-5	1.8E-6	6.4E-6	6.4E-6	3.9E-6	6E-7	4E-7	<1E-7	<1E-7
Channel efficiency *η'_n_*	4.4E-4	1.6E-4	3.1E-5	2.4E-4	1.6E-5	1.1E-5	1.4E-5	1.9E-6	6.2E-6	2.5E-7	3.7E-6	<1E-7	1E-7	<1E-7	<1E-7
**Delay *Δt_n_ [fs]***	**19**	**44**	**77**	**122**	**175**	**243**	**317**	**394**	**489**	**595**	**714**	**833**	**970**	**1120**	**1277**
**Delay uncertainty *δt_n_ [fs]***	**0.08**	**0.18**	**0.31**	**0.49**	**0.70**	**0.97**	**1.3**	**1.6**	**2.0**	**2.4**	**2.9**	**3.3**	**3.9**	**4.5**	**5.1**
